# TMS-evoked changes in brain-state dynamics quantified by using EEG data

**DOI:** 10.3389/fnhum.2013.00155

**Published:** 2013-04-25

**Authors:** Tuomas Mutanen, Jaakko O. Nieminen, Risto J. Ilmoniemi

**Affiliations:** ^1^Department of Biomedical Engineering and Computational Science, Aalto University School of ScienceEspoo, Finland; ^2^BioMag Laboratory, HUSLAB, Helsinki University Central HospitalHelsinki, Finland

**Keywords:** TMS, EEG, state space, brain dynamics, trajectory, recurrence quantification analysis

## Abstract

To improve our understanding of the combined transcranial magnetic stimulation (TMS) and electroencephalography (EEG) method in general, it is important to study how the dynamics of the TMS-modulated brain activity differs from the dynamics of spontaneous activity. In this paper, we introduce two quantitative measures based on EEG data, called mean state shift (MSS) and state variance (SV), for evaluating the TMS-evoked changes in the brain-state dynamics. MSS quantifies the immediate TMS-elicited change in the brain state, whereas SV shows whether the rate at which the brain state changes is modulated by TMS. We report a statistically significant increase for a period of 100–200 ms after the TMS pulse in both MSS and SV at the group level. This indicates that the TMS-modulated brain state differs from the spontaneous one. Moreover, the TMS-modulated activity is more vigorous than the natural activity.

## 1. Introduction

Combined transcranial magnetic stimulation (TMS) and electroencephalography (EEG) is able to probe the dynamics of the effective connectivity of the brain. Using TMS–EEG it has been possible to show how the activation induced on one hemisphere advances to the contralateral side (Komssi et al., 2002). TMS–EEG can also be used to study the effect of the brain state on the dynamics of excitability: Nikulin et al. (2003) showed that voluntary preparation for hand movement changes the EEG responses evoked by stimulating the primary motor cortex. Massimini et al. (2005) showed that changes in the state of consciousness affect the effective connectivity. Huber et al. (2013) studied the effect of lack of sleep on cortical excitability, demonstrating increased TMS-evoked EEG responses with prolonged wakefulness.

Furthermore, TMS can be used to modulate brain dynamics. Combined TMS–EEG studies have shown that even a single TMS pulse is able to induce changes in the frequency spectrum of brain activity (Paus et al., 2001; Fuggetta et al., 2005; Rosanova et al., 2009). Using preparatory repetitive TMS (rTMS), it has been possible to modulate subsequent single-pulse TMS–EEG responses. Van Der Werf and Paus (2006) showed that facilitatory rTMS at 0.6 Hz on the primary motor cortex had a significant increasing effect on the subsequent N45 deflections. Similarly, Esser et al. (2006) showed that by applying rTMS on the primary motor cortex at 5 Hz, it is possible to significantly potentiate single-pulse deflections with latencies of 15–55 ms. Recently, frequency-tuned rhythmic TMS has been shown to selectively bias perception (Romei et al., 2010) via entrainment of ongoing oscillatory activity (Thut et al., 2011).

However, the differences between pre- and post-TMS activity, i.e., how a single TMS pulse affects concurrent brain dynamics, have not been well characterized. One difficulty in analyzing changes from pre- to post-TMS brain state is that at the trial level the TMS-evoked changes are masked by spontaneous background activity. Furthermore, the spontaneous activity varies from one trial to another.

In this paper, we introduce two quantitative recurrence measures called mean state shift (MSS) and state variance (SV). They can be computed from unaveraged EEG signals and averaged afterwards to show possible changes in the brain dynamics due to TMS. The particular interest of the present work is to study the effects of TMS on the brain state. We use TMS–EEG data to show that TMS, indeed, has a significant effect on both MSS and SV.

## 2. Materials and methods

### 2.1. Connection between the brain state and TMS−EEG

**Figure 1:**
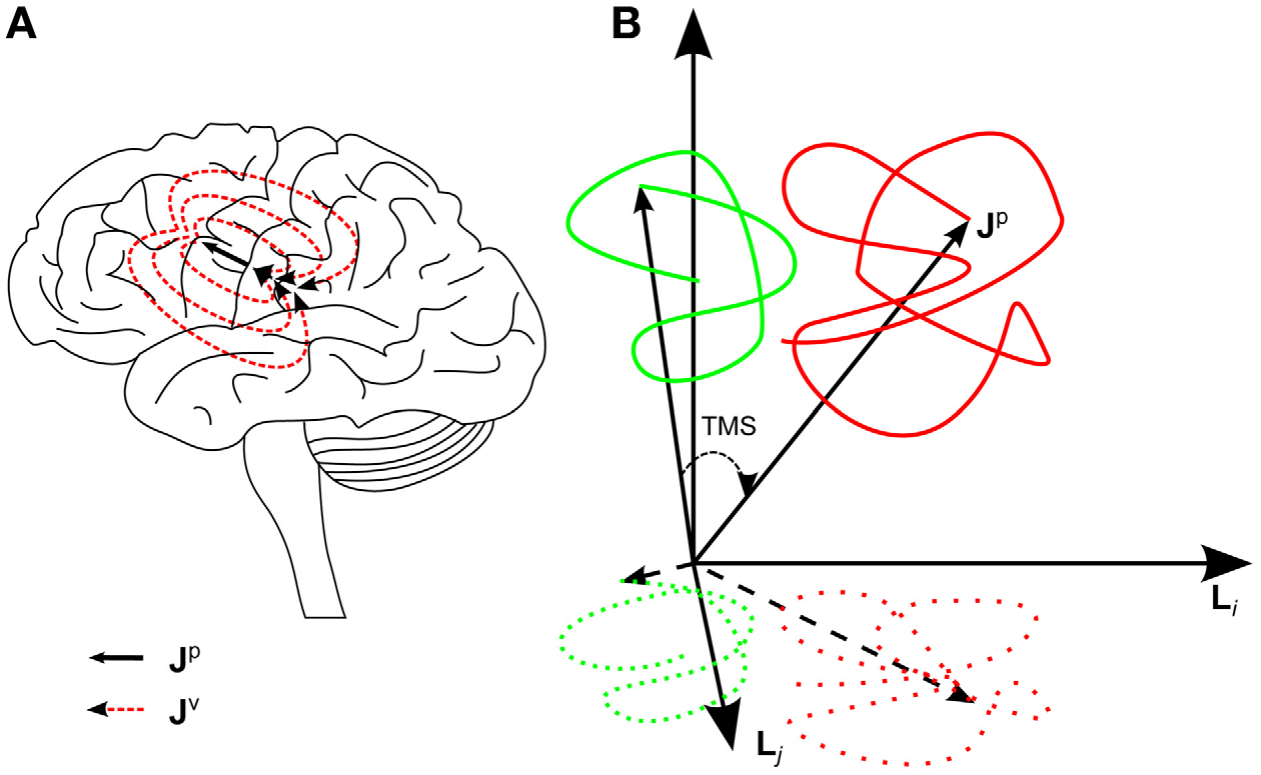
**(A)** The black arrow represents one primary current source, a flow of ions in synapses in the left primary motor cortex. The dashed red lines represent the returning volume current. **J**^p^(**r**) describes the whole primary current distribution in the brain. **(B)** A schematic image of the hypothesis concerning the effect of TMS on the brain state. The green and red curves correspond to pre- and post-TMS brain-state trajectories, respectively. The spontaneous activity draws a trajectory in a certain region. TMS shifts the brain state to a new region in the state space. Furthermore, the brain state fluctuates more after TMS because of the increased free energy until the state gradually returns to the original set of spontaneous states. The projection of these effects can be seen in the EEG signal space, spanned by channels *i* and *j*. In the signal space, the trajectories are measured only at discrete time points, which is emphasized with dotted curves.

The current distribution **J(r**, t) in the brain is often expressed in two parts:

(1) $$J(r,t)=J^{\text{p}}(r,t)+J^{\text{v}}(r,t),$$

where **J**^p^ is the primary current density arising from the bioelectric activation of neurons (e.g., post-synaptic currents), **J**^v^ is the volume current density, **r** is the position, and *t* is the time. **J**^v^ is passive, ohmic current density driven by **J**^p^ (Malmivuo and Plonsey, 1995) (Figure 1A).

**J**^p^ can be thought of as the primary source creating all the current density, which in turn affects the charge distribution that defines the electric potential. Hence, the EEG signal, i.e., the voltage, measured by channel *j* can be expressed as

(2) $$X_{j}(t)=\int L_{j}(r^{\prime })\cdot J^{\text{p}}(r^{\prime },t)dv^{\prime },$$

where **L**_*j*_**(r)** is the lead field determined by the geometry of the measurement set-up and the conductivity of the head (Ilmoniemi and Kičić, 2010). In other words, **L**_*j*_(**r**) describes how efficiently channel *j* detects primary current at **r**.

Thus, EEG can be considered the measurement of the projection of the primary current density on the signal space, the projection being defined by the lead fields of the channels. Since the primary current density describes accurately the electric state of the brain, the EEG signal can be considered a projection of the electric brain state. As **J**^p^(**r**, *t*), i.e., the state of the brain, changes, it draws a trajectory in the multidimensional state space. The trajectory is also projected on the EEG signal space (Figure 1B).

On the other hand, TMS can be used to modulate **J**^p^. In the brain, the changing magnetic field induces an electric field, which elicits action potentials in the axons. When action potentials reach synapses, post-synaptic currents that are visible in the EEG signal are created.

Our hypothesis is that TMS moves the brain higher in the energy landscape (here the energy landscape describes the tendency of the system to go from low probability to high probability states; from high energy to low energy) reflecting the information processing system. Hence the TMS-modulated activity at the stimulation site is seen in the brain state trajectory as a sudden shift to a new region in the state space (Figure 1B), which is spontaneously occupied only with a small probability. We test this hypothesis directly by measuring MSS, which quantifies the mean distance between the brain states from two different time intervals. According to the hypothesis, it is expected that due to TMS there is a transient increase in MSS with respect to the baseline.

If the state of the brain is higher in the energy landscape, it means increased free energy for the brain to act. The brain tends to minimize the free energy and get closer to some local energy minimum leading to enhanced fluctuation after TMS. This fluctuation is quantified using SV, which we expect to be increased until the system is closer to spontaneously probable states.

If the changes in the trajectory due to a single TMS pulse are large enough, they could also be visible in the obtained EEG signal. Since the EEG signal is a low-dimensional projection of the original primary current distribution, any significant difference between the signal vectors also indicates a difference in the original state vectors.

### 2.2. Data used

We used 16 TMS–EEG datasets from our database to characterize TMS-elicited changes in the brain activity. The datasets had been measured from healthy subjects (six males and four females; age varied between 24 and 28 years) who gave their written consent before the experiments. The measurements had been approved by the Ethics Committee of the Hospital District of Helsinki and Uusimaa and they followed the Declaration of Helsinki.

In all datasets, TMS stimuli were given with the same Nexstim eXimia system using a figure-of-eight coil with the outer loop diameter of 70 mm. The stimuli were targeted to the right hand area at the left primary motor cortex. Similarly, the TMS-compatible EEG device (Nexstim eXimia) was the same in all datasets. All the electrodes were prepared so that their impedances were below 5 kΩ. Additionally, two electrodes were attached close to the eyes to record ocular artifacts. The EEG sampling frequency was 1450 Hz.

The datasets were chosen based on the overall signal quality, i.e., low muscle- (Mutanen et al., 2012) and ocular-artifact (Ilmoniemi and Kičić, 2010) levels. The data acquisition and timing paradigms varied slightly across the analyzed datasets which ensures that our findings can be generalized over different measurement set-ups. The details of the measurement paradigms and exceptions are provided in Table 1.

**Table 1:** **The measurement parameters in different datasets**.

**Dataset**	**Stimulation target**	**Intensity [MT]%**	**Noise masking**	**ISI [s]**	**Number of stimuli**	**Coil type**
1	APB	100	Yes	2–3	100	monophasic
2	APB	100	Yes	2–3	100	monophasic
3	ADM	100	Yes	2–3	259	monophasic
4	APB	100	Yes	2–3	113	monophasic
5	APB	110	No	2–3	100	biphasic
6	APB	110	No	2–3	100	biphasic
7	APB	90	No	1, 3, or 5	376	monophasic
8	APB	90	No	1, 3, or 5	306	monophasic
9	APB	90	No	1, 3, or 5	326	monophasic
10	M1	<100	No	2–3	60	monophasic
11	ADM	100	No	2–3	89	monophasic
12	APB	100	Yes	2–3	115	monophasic
13	ADM	100	Yes	2–3	60	monophasic
14	ADM	100	Yes	2–3	60	monophasic
15	ADM	100	Yes	2–3	60	monophasic
16	APB	100	Yes	2–3	60	monophasic

Stimulation target refers to the cortical area controlling the named muscle (abductor pollicis brevis or abductor digiti minimi). In dataset 10, no hand muscle areas were found and stimulation was given to area usually responsible for controlling the right hand. The stimulus intensities are given with respect to the resting motor threshold (MT) intensity. When noise masking was given, it was adjusted until the subject reported to not hearing the click. Interstimulus interval (ISI) either varied randomly between 2 and 3 s or among 1, 3, and 5 s.

To see the possible changes more clearly, only 12 channels close to the stimulus location were used to form the signal subspace under study. Hence, only channels Fc_5_, Fc_3_, Fc_1_, Fc_*z*_, C_5_, C_3_, C_1_, C_*z*_, Cp_5_, Cp_3_, Cp_1_, and Cp_*z*_ according to the international 10–20 system were studied.

### 2.3. Computing mean state shift and state variance

Recurrence analysis was introduced by Eckmann et al. (1987) to qualitatively analyze state-space trajectories in order to characterize different dynamical systems. Recurrence analysis describes how often and for how long a certain physical state occurs. The basic idea is simple. An appropriate threshold is first chosen. If the distance between two states is smaller than the threshold value, the state vectors are considered to represent the same state. In EEG studies, recurrence analysis has been used to study, for instance, neurological disorders (e.g., Babloyantz, 1991; Pijn et al., 1997; Ouyang et al., 2008).

To provide quantitative results, several recurrence quantification analysis (RQA) measures, such as recurrence density, determinism, and entropy, have been introduced (Marwan et al., 2007). Strictly speaking, our measures do not fall under RQA category since we do not have any fixed threshold. Instead, we describe the obtained data by measuring average distances between state vectors. This is sometimes referred to as global recurrence (Marwan et al., 2007) or unthresholded recurrence analysis (Iwanski and Bradley, 1998; Marwan et al., 2007). However, the lack of a threshold value makes our measures more robust since one does not have to choose any arbitrary threshold. To our knowledge, RQA has not been previously applied to TMS–EEG data.

Let us now have a trajectory 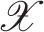 of a system drawn in the state space, or as in our case, drawn in the EEG signal space that is a projection of the original state space. The measured trajectory consists of signal vectors at discrete time points *t*_*l*_: 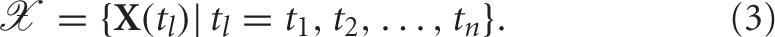


The signal vector at time *t*_*l*_ is defined as

(4) $$X(t_{l})={\left[X_{1}(t_{l}),X_{2}(t_{l}),\ldots ,X_{D}(t_{l})\right]}^{\text{T}},$$

where *D* is the dimension of the signal space, defined by the number of channels, and *X*_*j*_ is the signal measured by channel *j*, defined in Equation 2.

As the name implies, MSS describes the mean distance between state vectors belonging to two different time intervals:

(5) $$\text{MSS}\equiv \text{MSS}(T_{l},T_{k})=\frac{1}{N_{l}N_{k}}\sum_{t_{l}\;\in \;T_{l}}\;\sum_{t_{k}\;\in \;T_{k}}\|X(t_{l})-X(t_{k})\|,$$

where ǁ • ǁ is the Euclidean distance, and *T*_*l*_ and *T*_*k*_ are time intervals consisting of *N*_*l*_ and *N*_*k*_ discrete time points, respectively. Additionally, in this paper, *T*_*l*_ *∩ T*_*k*_ = ∅ and *N*_*l*_ = *N*_*k*_. The purpose of MSS is to show whether there is a more dramatic average change in the state due to TMS than due to the normal fluctuations in time. Hence, MSS quantifies the immediate effect of TMS on the brain state.

On the other hand, SV measures the rate at which the state changes during a given time interval. It is anticipated that the motion of the state would be more vigorous right after the TMS pulse than before it because of the locally higher free energy which the system tends to minimize. SV is defined as:

(6) $$\text{SV}\equiv \text{SV}(T_{l})=\frac{1}{N_{l}}\sum_{t_{l}\;\in \;T_{l}}\|X(t_{l})-\bar{X}(T_{l}){\|}^{2},$$

where

(7) $$\bar{X}(T_{l})=\frac{1}{N_{l}}\sum_{t_{l}\;\in \;T_{l}}X(t_{l}).$$

Conventionally, TMS-evoked potentials are made visible in the EEG by performing several trials and averaging the responses afterwards (e.g., Komssi et al., 2002; Massimini et al., 2005; Lioumis et al., 2009). This is done to suppress the background activity that masks the TMS-evoked potentials. However, it is difficult to design a method to average both the pre- and post-TMS intervals over trials to show the TMS-evoked changes in the activity. Therefore, pre- and post-TMS activity are ideally compared at the trial level. Unfortunately, the changes due to TMS at the trial level are subtle (Mäki and Ilmoniemi, 2010). One benefit of MSS and SV is that they can be computed from trial-level data and averaged later on to highlight the TMS-elicited changes.

### 2.4. Analyzing the effect of TMS on MSS and SV

Before any further data analysis, all the datasets were visually inspected. Bad EEG channels and any trials contaminated by ocular artifacts were removed. The data were also band-pass filtered to 2–80 Hz using a second-order Butterworth filter.

Both MSS and SV were calculated from unaveraged trial-level data. Each accepted trial from each dataset was divided into five different time intervals: *T*_1_ = [−200, −100], *T*_2_ = [−100, 0], *T*_3_ = [15, 115], *T*_4_ = [115, 215], and *T*_5_ = [215, 315], where the times are given in [ms] with respect to the moment of the TMS impulse (minus sign indicating time before the stimulus). Interval *T*_3_ started 15 ms after the stimulus to ensure that the small muscle artifacts (Mutanen et al., 2012) present in some datasets did not affect the results. Additionally, two time intervals, *T*_b1_ = [−400, −300] and *T*_b2_ = [−300, −200], were chosen for baseline scaling.

MSS was always calculated with respect to time interval *T*_1_. Hence, for each accepted trial from each dataset, four MSS values were obtained: MSS(*T*_1_, *T*_2_), MSS(*T*_1_, *T*_3_), MSS(*T*_1_, *T*_4_), and MSS(*T*_1_, *T*_5_). These MSS values were then averaged over trials for each dataset. The obtained averages were divided with the subject-dependent average baseline value, MSS(*T*_b1_, *T*_b2_), to suppress the differences in the subjects and to emphasize the changes due to different time intervals. The effect of time interval on MSS was studied using one-way ANOVA, with different subjects corresponding to different samples. After ANOVA, Bonferroni-corrected *post-hoc* tests were performed to compare the grand averages of the MSS values. To minimize the possibility that auditory artifacts contaminated the results, we performed the same analysis for all the data and only for datasets measured with noise masking.

SV was calculated for each time interval, providing five numerical values for each trial: SV(*T*_1_), SV(*T*_2_), SV(*T*_3_), SV(*T*_4_), and SV(*T*_5_). The same analysis, including averaging, baseline scaling, and statistical testing described above for MSS was also applied to SV. In this case, the baseline division was done using SV(*T*_b2_), again for each dataset individually.

## 3. Results

**Figure 2:**
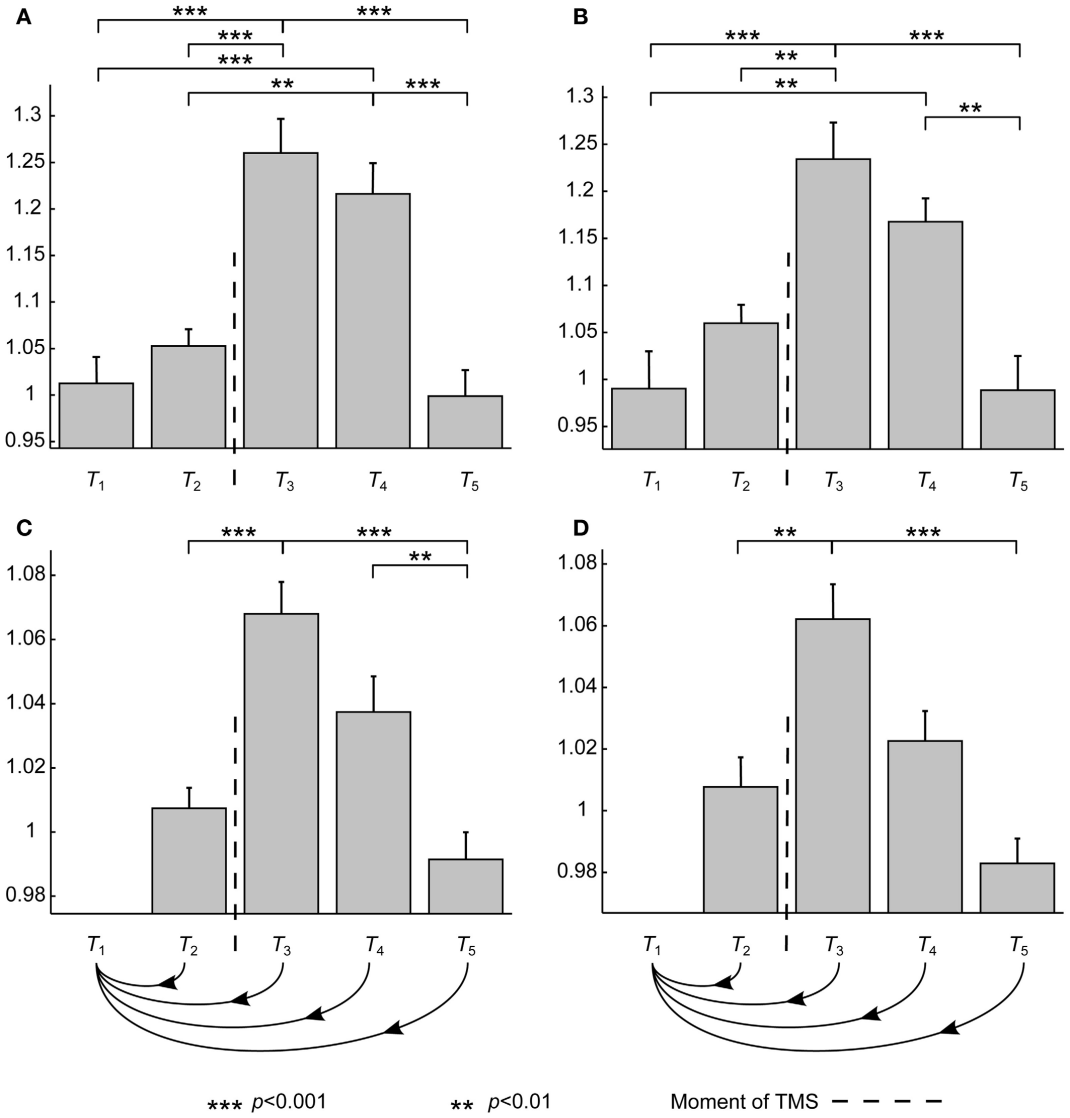
**The results averaged over subjects.** The asterisks show statistically significant differences between different conditions after the *post-hoc* tests. Error bars show ± standard-error-of-the-means calculated over datasets. Vertical axes are dimensionless and show the differences with respect to the baseline. *T*_1_, *T*_2_, *T*_3_, *T*_4_, and *T*_5_ refer to time intervals (in ms) [−200, −100], [−100, 0], [15, 115], [115, 215], and [215, 315], respectively. **(A,B)** SV at different time intervals averaged over all datasets and averaged over only those with noise masking, respectively. **(C,D)** MSS at different time intervals averaged over all datasets and averaged over only those with noise masking, respectively. The curvy arrow lines indicate that, with MSS, all the time intervals are compared to *T*_1_.

TMS seemed to have the anticipated effects: Both MSS and SV were increased (Figure 2). ANOVA showed that the time interval had a significant effect on SV (*p* < 0.001). Furthermore, *post-hoc* tests revealed a significant increase in SV during time intervals *T*_3_ and *T*_4_ compared to SVs measured at the other time intervals (Figure 2A). SV(*T*_3_) and SV(*T*_4_) were 20–25% higher than the baseline value, SV(*T*_b2_).

To show that the observed changes were not due to auditory responses, similar analysis was performed over only those datasets measured with auditory masking. ANOVA showed the same significance level for the effect of time interval (*p* < 0.001). Also the *post-hoc* test results were qualitatively very similar (Figure 2B). In general, only the significance levels had moderately increased, except that there was no more a statistically significant difference between SV(*T*_2_) and SV(*T*_4_).

Also in the case of MSS, the time interval had an overall significant effect (*p* < 0.001). Furthermore, *post-hoc* tests showed that the MSS value right after the stimulus was significantly increased when compared to MSS(*T*_1_, *T*_2_) and MSS(*T*_1_, *T*_5_) (*p* < 0.001) (Figure 2C). With MSS(*T*_1_, *T*_3_) and MSS(*T*_1_, *T*_4_), an increase of 4–6% was observed when compared to the baseline value, MSS(*T*_b1_, *T*_b2_). With MSS, the only difference between the complete data analysis and the analysis done over auditory-masked data was that in the latter no statistically significant difference between MSS(*T*_1_, *T*_4_) and MSS(*T*_1_, *T*_5_) could be obtained regardless of the large difference in the grand averages. In addition, the significance level for the difference between MSS(*T*_1_, *T*_2_) and MSS(*T*_1_, *T*_3_) had increased (*p* < 0.01) (Figure 2D).

The results were not as uniform at the subject level although 13 datasets showed an increase in both SV and MSS due to TMS. However, the durations of the effects differed between subjects. In most cases, the effects lasted 100–200 ms, but in a few cases the measures did not return to the baseline level. Additionally, the sizes of the changes varied significantly: In SV, the increase was 5–50% depending on the dataset, whereas in MSS the increase was 5–15%.

## 4. Discussion

Our results show that the measures introduced in this work are able to reveal differences in brain dynamics. The grand averages showed a significant increase both in SV and in MSS after TMS until they returned back to the baseline level. However, between the datasets, one could observe some variation even though 13/16 datasets showed an increase in MSS and SV due to TMS.

In the future, the presented measures should be applied to more homogeneous data to see whether the changes at the subject level would be repeatable. However, our hypothesis concerning the effects of TMS on the brain state relates to TMS in general. Thus, the use of datasets with moderate differences is, in this sense, justified.

Both SV and MSS could be easily applied to some other event-related-potential studies where the method to change **J**^p^(**r**) would differ from TMS. Furthermore, the connection between the brain state and the EEG signal space is completely analogous to magnetoencephalography (MEG) signal space (Ilmoniemi and Williamson, 1987; Uusitalo and Ilmoniemi, 1997), only the lead field presented in Equation 2 would be different. Thus, SV and MSS would be directly applicable to MEG data. Based on our results, RQA tools seem promising in studying the brain dynamics affected by any stimulation.

The increase in MSS implies that the brain activation following TMS occupies different regions in the brain state space than spontaneous activity. Although numerous empirical results (e.g., Komssi et al., 2002, 2004, 2007; Massimini et al., 2005; Lioumis et al., 2009) lead to expect that TMS changes the primary current distribution, it is far from self-evident that the sudden shift would be measurable from trial-level EEG data, given that EEG is an extremely low-dimensional projection of the original brain state. As discussed earlier, here conventional averaging is not an option. Indeed, the results show that, although the changes in MSS were statistically significant, they were still quite subtle, which is not a surprise, since the primary activation due to TMS is very focal (Hannula et al., 2005). Thus, most of the background activity is likely to stay similar even after the stimulus.

The increase in SV indicates that TMS-modulated activity differs in nature from spontaneous activity. In grand averages, there were differences of up to 25% between pre-TMS SVs and post-TMS SVs, implying that TMS-modulated activity proceeds faster in the state space than the spontaneous one.

Because this study was based on analyzing data measured earlier for other purposes, we lacked sham-TMS data. Hence, we cannot completely exclude the possibility that the increases in SV and MSS are partially due to somatosensory or auditory responses. Indeed, white noise was delivered to the subjects' ears (Paus et al., 2001) to minimize the auditory response at ~100 and ~180 ms in only some of the datasets. Thus, especially the analysis of datasets 5–10 might be affected by the auditory response (Nikouline et al., 1999). The somatosensory response due to scalp nerve activation is likely to have a smaller contribution to the observed changes, since the studied channels were located close to the stimulation site and the somatosensory responses from the scalp are seen on the contralateral hemisphere (Bennett and Jannetta, 1980; Hashimoto, 1988). However, in the future, sham-TMS measurements would be useful to quantify the auditory and somatosensory artifacts in MSS and SV.

Since the stimulation intensity was in all datasets around 100% of the motor threshold, we have to consider the possibility that the motor-evoked-potential (MEP)-related peripheral somatosensory signal might have contributed to the studied measures. Although Nikulin et al. (2003) showed that the MEP-related sensations did not significantly affect the average TMS-evoked EEG responses, it would be advisable to conduct the analysis described in the present work over data measured when TMS has been delivered with sub-threshold intensity or to a non-motor area to ensure that MSS and SV are not affected considerably by the tactile sensation of a MEP.

In the present work, we did not study the dynamical changes in solely spontaneous EEG data. However, we are convinced that the changes in MSS and SV are due to TMS (and indeed possibly due to sensory stimuli elicited by the magnetic impulse) since the increase in SV or MSS is short-lived and returns back to baseline levels.

The effects of TMS on SV and MSS seemed to last 100–200 ms. However, the length of the studied time intervals was 100 ms, limiting the temporal resolution. In principle, the temporal resolution could be improved simply by reducing the length of the time intervals. Unfortunately, this is likely to decrease the signal-to-noise ratio of the measures.

The changes in SV and MSS, in a broad sense, can be explained with the second law of thermodynamics. Although there is a substantial physiological system constantly providing energy and information to the brain, we can approximately consider the brain as an isolated system for the short period of time (~300 ms) that we measure it after the impulse. The spontaneous state before the TMS impulse lies relatively low in the free-energy landscape. The large impulse changes the brain state to a new state that normally has a lower probability meaning increased free energy. The observed activation following the impulse is partially due to the brain settling itself again to a lower energy level. Similar ideas have also been presented earlier (e.g., Hopfield, 1982; Friston et al., 2006; Friston, 2010), although the earlier article discusses the free energy of an artificial neural network and the latter articles deal free energy of a system in a more general level. In short, the results can be interpreted as follows: (1) With TMS, we do work to change the state of the brain, which can be seen in MSS. (2) The brain minimizes the locally high free energy due to work done by TMS, which can be seen as increased SV.

In conclusion, we introduced two novel quantitative tools that were able to characterize dynamic differences between spontaneous and TMS-modulated activity. The results might help us better understand the mechanisms of TMS and combined TMS–EEG method in general.

### Conflict of interest statement

Risto J. Ilmoniemi is an advisor and a minority shareholder of Nexstim Ltd. The other authors declare that the research was conducted in the absence of any commercial or financial relationships that could be construed as a potential conflict of interest.
